# Clinical Practice Guidelines for the Management of Behavioral and Psychological Symptoms of Dementia: A Systematic Review With AGREE II

**DOI:** 10.3389/fneur.2022.799723

**Published:** 2022-05-25

**Authors:** Huixuan Ma, Xinliang Lu, Aihong Zhou, Fen Wang, Xiumei Zuo, Minmin Zhan, Qi Zou, Shuting Gong, Yufei Chen, Jihui Lyu, Longfei Jia, Jianping Jia, Cuibai Wei

**Affiliations:** ^1^Innovation Center for Neurological Disorders, Xuan Wu Hospital, Capital Medical University, Beijing, China; ^2^Department of Neurology, Xuan Wu Hospital, Capital Medical University, Beijing, China; ^3^Department of Neurology, Beijing Friendship Hospital, Capital Medical University, Beijing, China; ^4^School of Biological Science and Medical Engineering, Beihang University, Beijing, China; ^5^Department of Cooperation of Chinese and Western Medicine Institute, Changchun University of Traditional Chinese Medicine, Changchun, China; ^6^Beijing Geriatric Hospital, Beijing, China

**Keywords:** dementia, behavioral symptoms, practice guideline, antipsychotic agents, drug therapy

## Abstract

**Background:**

High-quality clinical practice guidelines (CPGs) are important for the effective treatment of behavioral and psychological symptoms of dementia (BPSD). However, recommendations provided by different quality guidelines may lead to varied clinical practice outcomes.

**Objective:**

To assess the quality of available CPGs for the management of BPSD and summarize the best recommendations for treating BPSD.

**Methods:**

This was a systematic review of CPGs for the management of BPSD with data obtained from electronic databases and evaluated using the Appraisal of Guidelines for Research and Evaluation II instrument, consisting of six domains: “Scope and purpose”, “Stakeholder involvement”, “Rigor of development”, “Clarity of presentation”, “Applicability”, and “Editorial independence”. The criteria for high-quality guidelines were set as: the score of high-quality guidelines in the “Rigor of development” domain should be ≥60% and as well as a score of >60% in at least three other domains. High-quality guidelines were selected for recommendation extraction, and the final recommendations were formed in combination with the latest meta-analysis and randomized clinical-trial results.

**Results:**

In term of median scores in each domain for the six included CPGs, “Scope and purpose” (87.5%) scored better than all others, whereas “Applicability” (46.5%) was the domain with the lowest score. Four CPGs (2015 APA, 2018 NICE, 2018 CANADA, 2020 EAN) met the criteria of high-quality guidelines and were used to extract recommendations. From these four CPGs, nine specific recommendations related to the management of BPSD were summarized, of which seven were related to pharmacological treatment and two to non-pharmacological treatment. These recommendations covered the applicability of antipsychotic drugs, medication recommendations, withdrawal times, and several suitable non-pharmacological therapies.

**Conclusion:**

The quality of CPGs for the management of BPSD requires improvement, especially for the “Applicability” domain. For psychotic-like symptoms in dementia, the use of antipsychotics should be based on the individual's risk-benefit ratio, and the use of atypical antipsychotics seems to be a better choice. Non-pharmacological treatments may be suitable for emotional symptoms and sleep disorders.

**Systematic Review Registration:**

https://www.crd.york.ac.uk/prospero/display_record.php?ID=CRD42020209204.

## Introduction

Dementia has a higher prevalence among older individuals and currently affects 50 million patients worldwide, a number which is expected to increase to 152 million by 2050, according to the 2019 Alzheimer's disease International report (1). Meanwhile, more than 90% of patients experience behavioral and psychological symptoms of dementia (BPSD), including agitation, apathy, depression, repetitive questioning, psychosis, aggression, sleep problems, wandering, and various inappropriate behaviors (2). These symptoms are associated with decline in quality of life, poor functional status, and worsened cognition (3, 4). Therefore, the management of BPSD is of high medical and social priority. To assist physicians and patients in making decisions regarding the appropriate management of BPSD, different countries and regions have developed their own clinical practice guidelines (CPGs) through systematic reviews and assessments of the benefits and harms of different treatment options. However, the multiple available guidelines often lead to uncertainty for clinicians regarding which CPGs should be used to treat BPSD. Our work primarily aimed to synthesize high-quality evidence-based CPGs available for the management of BPSD. The secondary aim was to identify domains of therapeutic concern within the existing guidelines that can be further researched to improve future guideline development.

## Materials and Methods

The protocol of this systematic review was registered in the International Prospective Register of Systematic Reviews, with number CRD42020209204. This review was conducted in strict accordance with the guidelines of Preferred Reporting Items for Systematic Reviews and Meta-analyses.

### Data Sources and Searches

**Figure 1:**
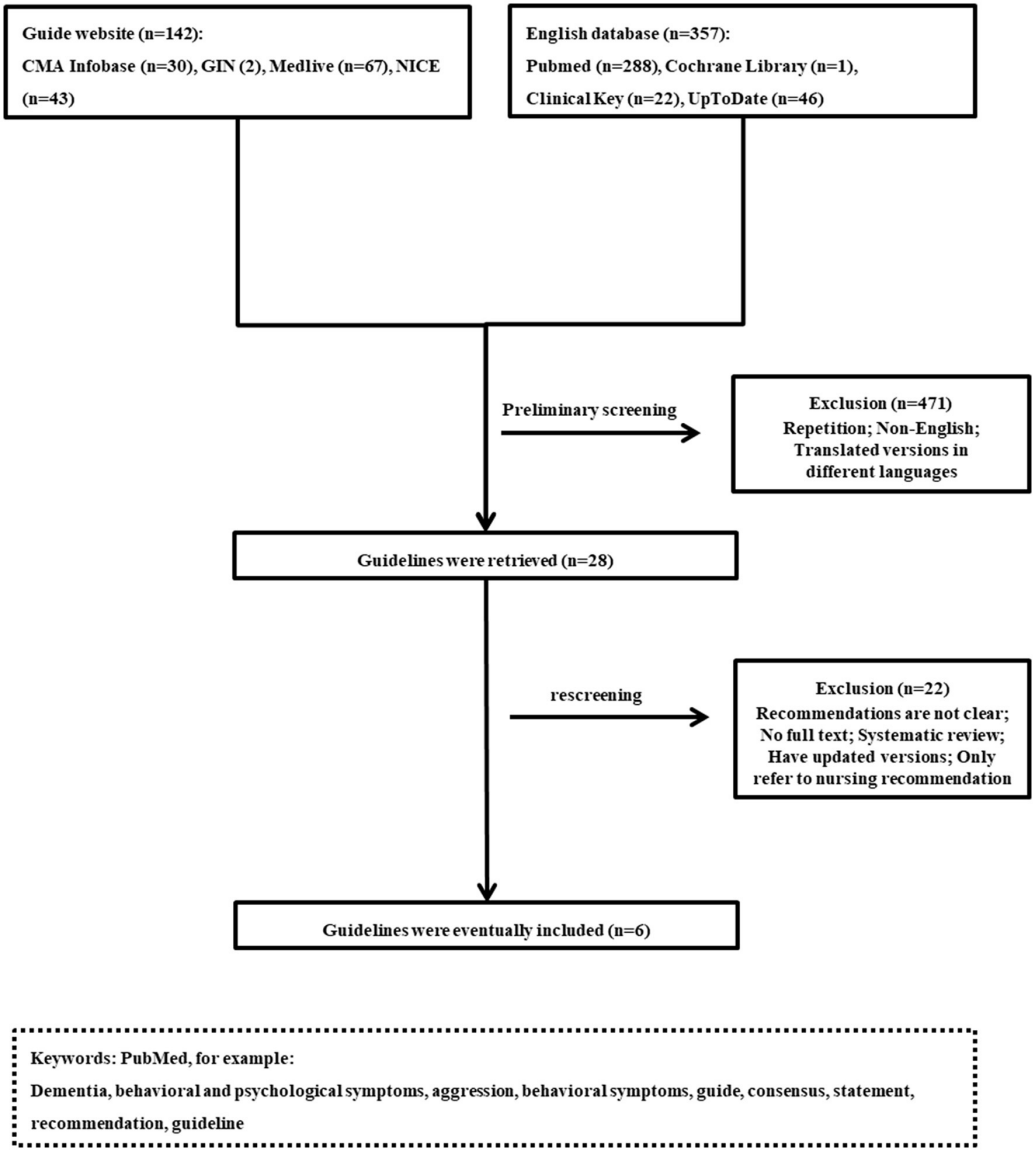
The flowchart of the systematic search. (CMA Infobase), Canadian Medical Association Infobase; (GIN), Guidelines International Network; (NICE), National Institute for Health and Care Excellence.

Two independent researchers conducted investigations using the following electronic bibliographic databases: PubMed, Cochrane Library, ClinicalKey, and UpToDate; as well as guideline specific sources: Canadian Medical Association Infobase, Guidelines International Network, National Institute for Health and Care Excellence (NICE), and Medlive. These were used to search for recent CPGs for the management of BPSD. We searched with terms such as “dementia”, “behavioral and psychological symptoms”, “behavioral symptoms”, “psychiatric symptom”, “guideline”, and combinations. Azermai et al. (5) used the Appraisal of Guidelines for Research and Evaluation II (AGREE II) (6) tool to evaluate the quality of guidelines related to the management of BPSD from 2003 to 2012. We conducted the search for guidelines published from January 2012 to August 2020, and only in the English language. A flowchart of the search is shown in Figure 1.

### Study Selection

First, the obtained results were imported into EndNote X9 to remove duplications. Then, studies were included after primary screening (read title and abstract) and secondary screening (read full text) of the above results. If any disagreement regarding whether to include an article occurred between the two independent reviewers, a third experienced review expert would be consulted.

We only included the most recent CPGs of international consensus, defined as documents developed by a nationally recognized committee, publicly funded institution, or medical society, providing recommendations for the management of BPSD, especially with respect to pharmacological treatment.

We excluded: (1) direct translations or interpretations of other guidelines; (2) documents where the full text was not available; (3) guidelines that solely provided recommendations on care; and (4) systematic reviews, letters to the editor, or frameworks of guideline development.

### Quality Assessment

The quality of the screened guidelines was assessed using AGREE II, which is a widely used guideline evaluation tool (composed of 23 items within six domains) (6). AGREE II consists of six domains. The six domains are “Scope and purpose”, “Stakeholder involvement”, “Rigor of development”, “Clarity of presentation”, “Applicability”, and “Editorial independence”. Each domain is assessed based on several “items”, with a total of 23 items. Each item is graded on a seven-point scale, with score of one indicating that the guideline does not comply with this item at all, and a score of seven indicating that the guideline fully complies with this item. The higher the score, the higher the compliance degree of the guideline in this item. Details of the items are available online (http://www.agreetrust.org/). The median score of the CPGs in each domain is an important indicator of the overall performance of the guidelines; hence, we calculated the median score of the guidelines in each domain. For screening high-quality guidelines, the “Rigor of development” domain was evaluated first. The score of the CPGs in the “Rigor of development” domain should be ≥60% (5, 7), and as well as a score of >60% in at least three other domains. Guidelines that did not meet these criteria were removed from further analyses.

Prior to the scoring process, the two reviewers conducted the training exercises available on http://www.agreetrust.org/. The scoring consistency between the two reviewers was computed using the intraclass correlation coefficient (ICC) with a two-way random effects model for each domain using SPSS software (version 26.0, IBM Corp., Armonk, NY). The ICC levels of consistency were categorized based on their respective scores in four classes: poor (<0.40), fair (0.40–0.59), good (0.60–0.74), or excellent (0.75–1.00) (8). If there were differences between the scores of the two reviewers, a third appraiser was consulted.

### Data Extraction and Recommendation Generation

The two independent reviewers conducted information extraction on the six guidelines that passed the “Study Selection” criteria. During this process, one of the reviewers independently extracted data from the six CPGs using a standardized form in MS Excel 2010 (Microsoft Corp., Redmond, WA). Target information included the title, year of publication, developers, country, target population, and Grade of Recommendations Assessment, Development and Evaluation (9) was used to evaluate the quality of each guideline (see Table 1). Then, the recommendations for the management of BPSD were extracted from the selected high-quality guidelines, along with the strength of recommendations and evidence supporting recommendations. The management of BPSD was the focus of each guideline. We extracted recommendations for the treatment of symptoms addressed by the guidelines, such as psychotic-like symptoms, emotional symptoms, and sleep disorders. Specific practice recommendations for BPSD were also classified into pharmacological and non-pharmacological interventions. Finally, the second reviewer inspected the extraction results. For the recommendations extracted from the four high-quality guidelines, we analyzed the consistency between the guidelines for the same recommendation, combined with the latest meta-analysis and randomized clinical trial results to form our final recommendations.

**Table 1:** Clinical practice guideline characteristics and development methods for recommendations.

**Guideline**	**Relevant content**	**Developers**	**Country/region**	**Target population**	**Type of evidence**	**Grading system**
2015; APA	Use of antipsychotic medications when agitation or psychosis occurs in association with dementia	American Psychiatric Association	USA	Patients with dementia exhibiting agitation or psychosis	Randomized controlled trials, Systematic review, Expert opinion, Observational research, Patient values and preferences	YES
2018; DELPHI	Existing and emerging treatments for BPSD in Alzheimer's disease overall, as well as specifically for agitation and psychosis	Foreign expert group on psychiatry	International consensus panel	Patients with Alzheimer's disease exhibiting behavioral and psychological symptoms	Expert opinion, randomized controlled trials	NO
2018; IPS	Suggestions for the management of dementia	Indian Psychiatric Society	India	Patients with dementia	-	NO
2018; CANADA	When and how to safely taper and stop antipsychotics	Foreign expert group on psychiatry	Canada	Patients with behavioral and psychological symptoms of dementia and insomnia	Systematic reviews, expert consensus, randomized controlled trials	YES
2018; NICE	How dementia should be assessed and diagnosed	National Institute for Health and Clinical Excellence	United Kingdom	Patients with dementia	Systematic Reviews,Systematic review and Meta-analysis, Randomized controlled trials	YES
2020; EAN	Use of antipsychotics in dementia	European Academy of Neurology	Europe	Patients with dementia	Randomized controlled trials	YES

## Results

### Systematic Search and Screening of CPGs

In total, 28 results were screened from 499 total results based on title and abstract information. After analyzing the full text, six CPGs met the inclusion criteria. The characteristics of these six CPGs are listed in Table 1. These CPGs were developed by the clinical and scientific bodies of the United States, European Union, Canada, United Kingdom, India, and an international consensus panel composed of individuals from different countries (including Europe, the United States, United Kingdom, Australia, and Canada); we termed them 2015 American Psychiatric Association (APA) guideline (10), 2018 DELPHI consensus (11), 2018 IPS guideline (12), 2018 CANADA guideline (13), 2018 NICE guideline (14), 2020 European Academy of Neurology (EAN) guideline (15).

### Quality Appraisal of CPGs

#### Scope and Purpose

Domain 1 “Scope and purpose” assesses whether a guideline describes the overall purpose clearly, that is, whether a guideline describes its potential impact on patients and society when implemented to the specific clinical symptoms. Among the six included guidelines, the score of domain 1 was >60%, and the median score of domain 1 was the highest (87.5%) compared with those of the other five domains, suggesting that the guideline developers clearly elaborated on the overall purpose of the guideline (Table 2).

**Table 2:** Quality appraisal of dementia guidelines with the AGREE II instrument.

**Included guidelines**	**Scope and** **purpose**	**Stakeholder** **involvement**	**Rigor of** **development**	**Clarity and** **presentation**	**Applicability**	**Editorial independence**
2015 APA	94	75	79	92	63	96
2018 DELPHI	83	47	26	78	19	92
2018 IPS	75	6	19	72	33	0
2018 CANADA	92	86	81	92	71	83
2018 NICE	94	72	68	89	60	71
2020 EAN	78	58	60	72	29	75
Median	87.5	65	64	83.5	46.5	79

#### Stakeholder Involvement

Domain 2 “Stakeholder involvement” considers basic information regarding the guideline developers and the degree of participation of the audience in the guideline development process. Of the six included guidelines, four guidelines (2015 APA, 2018 NICE, 2018 CANADA, 2020 EAN) scored >60% in this domain, while two guidelines, 2018 DELPHI and 2018 IPS, scored low at 47% and 6%, respectively, because these two guidelines did not consider the audience's views through questionnaires or other methods during the guideline development process (item 5) and 2018 IPS did not provide basic information of the guideline developers (item 4).

#### Rigor of Development

In many studies, the score of domain 3 “Rigor of development” has been used as a criterion for assessing whether a guideline is of high quality. This domain requires the guideline to clarify the criteria for evidence retrieval and the process for developing recommendations and whether the guideline has undergone an external review process. Among the six included guidelines, four guidelines (2015 APA, 2018 NICE, 2018 CANADA, and 2020 EAN) scored >60%. Similar to domain 2, the score of two guidelines (2018 DELPHI and 2018 IPS) was low (26 and 19%, respectively). These guidelines did not address the selection criteria for evidence and were not submitted for external review prior to publication (items 8, 9, and 13).

#### Clarity and Presentation

Domain 4 “Clarity and presentation” specifies that the recommendations in the guideline are clear and legible. All six guidelines scored >60% in this domain. Furthermore, a high median score (83.5%) in domain 4 suggests that the guideline developers provide clear recommendations.

#### Applicability

Domain 5 “Applicability” requires the guideline to describe the factors that may facilitate and hinder the application of the guideline, as well as potential resource investment issues. The median score (46.5%) was the lowest among all domains. Of the six included guidelines, three guidelines (2015 APA, 2018 NICE, and 2018 CANADA) scored ≥60%, while the other three scored <60%, and the 2018 DELPHI scored 19%. The main reason for the low score is that this guideline does not consider the potential resource implications of applying the recommendations (item 20).

#### Editorial Independence

Domain 6 “Editorial independence” prescribes that the guideline should clearly state whether the interest of the funding has influenced the guideline development process and whether there is conflict of interest among the members of the development team. The median score was 79% in this domain, and the scores of five guidelines were >60%. 2018 IPS scored 0% because this guideline did not specify the requirements of this domain (items 22 and 23).

#### Overall Assessment

Among the six included guidelines, the median score for the six domains ranged from 46.5% to 87.5%, with five domains >60%. The above results showed excellent inter-rater reliability of the guidelines between two reviewers (Table 3). Four guidelines (2015 APA, 2018 NICE, 2018 CANADA, and 2020 EAN) met the definition of high-quality CPGs and were used to extract recommendations.

**Table 3:** Results of inter-rater reliability (ICC) for each guideline.

**Included guidelines**	**ICC**	**95% CI**	**F**	**P**
2015 APA	0.807	0.570–0.916	10.922	<0.001
2018 DELPHI	0.920	0.808–0.966	27.398	<0.001
2018 IPS	0.876	0.730–0.946	14.576	<0.001
2018 CANADA	0.828	0.641–0.923	10.737	<0.001
2018 NICE	0.884	0.425–0.964	29.705	<0.001
2020 EAN	0.851	0.679–0.934	11.913	<0.001

*CI, confidence interval*.

### Recommendation Extraction From High-Quality CPGs

The recommendations regarding the management of BPSD were extracted from the four high-quality CPGs (2015 APA, 2018 NICE, 2018 CANADA, and 2020 EAN). First, the content was divided into pharmacological and non-pharmacological treatments and further classified based on common symptoms including psychotic-like symptoms, emotional symptoms, and sleep disorders (see Supplementary Table 1). Then, this review sought the consistency of recommendations from Supplementary Table 1 and formed a recommendation sheet (Box 1) based on the latest meta-analysis and other randomized clinical trial results. This sheet contains nine recommendations, of which seven are related to pharmacological treatment and two to non-pharmacological treatment. These recommendations cover the applicability of antipsychotic drugs, medication recommendations, withdrawal times, and several suitable non-pharmacological therapies.

Box 1 **Summary of the recommendations for the management of BPSD.**

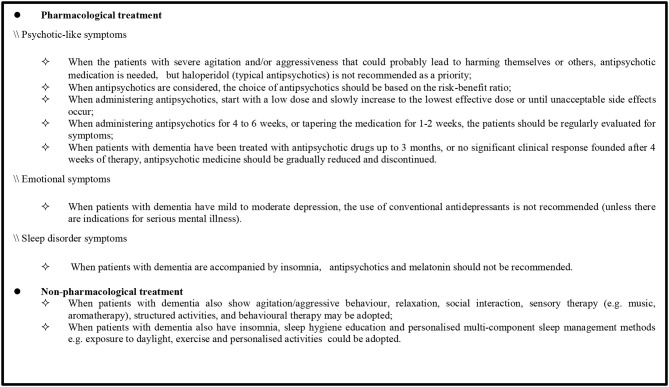

#### Pharmacological Treatment

##### Psychotic-Like Symptom Management

Antipsychotics are frequently used for the management of psychotic-like symptoms. In the four high-quality CPGs, applicability of antipsychotic drugs, medication recommendations, and withdrawal times are mentioned. Three guidelines (2015 APA, 2018 NICE, and 2020 EAN) recommend that antipsychotic medication should be used for patients who are severely agitated, aggressive, and/or cause harm to themselves or others. When using antipsychotics, the dose should start at the lowest level and slowly increase to the effective dose or until unacceptable side effects occur. In addition, the risk-benefit ratio of each drug should be considered when selecting an appropriate antipsychotic, as recommended in two guidelines (2015 APA and 2018 NICE).

Presently, both typical and atypical antipsychotics are commonly used for the treatment of psychotic-like symptoms. In the four high-quality CPGs, two guidelines (2015 APA and 2020 EAN) did not endorse the preferred use of haloperidol, a typical antipsychotic. The 2015 APA guideline recommends that in the absence of delirium, haloperidol should not be used as a first-line agent for nonemergency antipsychotic medication treatment. There was a weak recommendation in the 2020 EAN guideline that modern (atypical) antipsychotics can be used instead of haloperidol when pharmacological treatment of agitation/aggressive behavior is necessary. Conversely, risperidone, an atypical antipsychotic drug, is recommended in two guidelines (2018 CANADA and 2020 EAN). The 2018 CANADA guideline recommends that risperidone should be considered if BPSD relapse and antipsychotic treatment needs to be restarted. Moreover, risperidone could be considered as first-line treatment when pharmacological treatment of agitation/aggressive behavior is necessary, as suggested in the 2020 EAN guideline.

Antipsychotics should be considered for reduction or withdrawal in patients using antipsychotics when their symptoms have improved or serious side effects occur. The 2015 APA guideline recommends that dose reduction or discontinuation of antipsychotics should be considered in patients with agitation or psychosis who do not experience a clinically significant response after a 4-week trial of adequate dose of antipsychotics. Additionally, the 2018 CANADA guideline recommends that tapering and discontinuing antipsychotics slowly in collaboration with the patients and caregivers for patients with BPSD treated for at least 3 months, unless the patient experienced a recurrence of symptoms after prior attempts of tapering the antipsychotic medication. Even if symptoms recur, re-attempts to cancel the prescription within 3 months and at least two attempts to discontinue should be considered. Furthermore, periodic evaluation of symptoms is also necessary after initiation of antipsychotic medication, which will determine whether the treatment is effective or there are withdrawal symptoms. During the application of antipsychotics, two guidelines (2018 NICE and 2015 APA) recommend that patients be assessed for symptoms at least monthly or every 6 weeks to check whether they still require the medication. In the phase of tapering antipsychotics, the 2018 CANADA guideline encourages close monitoring for withdrawal symptoms in patients, especially in those with severe baseline BPSD or long-standing use of antipsychotics, every 1 to 2 weeks. After drug withdrawal, the 2015 APA guideline recommends that evaluation be performed for at least 4 months to identify signs of recurrence.

##### Management of Emotional Symptom and Sleep Disorder

For the management of emotional symptoms, the use of antidepressants is mentioned in the 2018 NICE guideline; it recommends that antidepressants should not be routinely offered to manage mild to moderate depression in individuals with mild to moderate dementia, unless they are indicated for a pre-existing severe mental health problem.

In the management of sleep disorders, two guidelines (2018 NICE and 2018 CANADA) make recommendations for the use of melatonin and antipsychotic medications, respectively. The 2018 NICE guideline recommends that melatonin should not be offered to manage insomnia in individuals with Alzheimer's disease. The other guideline (2018 CANADA) indicates that the antipsychotic use for the treatment of insomnia should be eliminated based on the lack of evidence for the efficacy of antipsychotics in treating insomnia, potentially harmful side effects, and high cost of treatment.

#### Non-pharmacological Treatment

Regarding the non-pharmacological treatment of BPSD, two guidelines (2018 CANADA and 2018 NICE) recommend methods for patients with dementia and agitation, mild to moderate depression, or sleep disorders. When patients with dementia show agitation/aggressive behavior, the 2018 CANADA guideline recommends non-pharmacological interventions such as relaxation, social interaction, sensory therapy (music, aromatherapy), structured activities, behavioral therapy, and indicates that attention should be paid to improve the patient's surrounding environment (e.g., light and noise). Meanwhile, the 2018 NICE guideline recommends ensuring that patients with dementia should participate in psychosocial and environmental interventions for distress during antipsychotic treatment and drug discontinuation. When patients with dementia also show emotional symptoms, 2018 NICE recommends that those who have mild to moderate dementia and mild to moderate depression and/or anxiety should receive psychological treatment. When patients with dementia have sleep disorders, 2018 NICE recommends methods such as personalized multicomponent sleep management, including sleep hygiene education; 2018 CANADA also refers to this.

## Discussion

### Quality Appraisal of CPGs

This study used AGREE II to evaluate the quality of guidelines for the management of BPSD in six domains, i.e., “Scope and purpose”, “Stakeholder involvement”, “Rigor of development”, “Clarity and presentation”, “Applicability”, and “Editorial independence”, and found that the median scores in all domains were >60% except for the “Applicability” domain (Table 2). The “Scope and purpose” and “Clarity and presentation” domains had the highest scores at 87.5% and 83.5%, respectively, possibly because all guidelines clearly elaborate on the purpose and explicitly express recommendations related to the management of BPSD. As for the “Editorial independence” domain, most guidelines refer to “what impact the funding institutions had on the development process of the guidelines” in the text, resulting in high scores (79%) in this domain. This suggests that explaining the conflict of interests among the members of the development team will be key to guaranteeing a high score in this domain. Compared with the above three domains, the scores of “Stakeholder involvement” and “Rigor of development” were lower, at 65% and 64% respectively. The reason for the low score in the domain of “Stakeholder involvement” was that two guidelines (2018 IPS and 2020 EAN) scored low in this domain, which may be related to the fact that the guidelines do not mention basic information of the makers and not consider the intentions of the patients. Therefore, a comprehensive description of the information of the makers of the guidelines and the use of questionnaires and other methods to fully consider the intention of the patients will help improve the score in this domain. In the domain of “Rigor of development”, two guidelines (2018 DELPHI, 2018 IPS) did not use standard methodological tools to evaluate the literature evidence on which the recommendations were based, resulting in scores <60%. Therefore, the application of a systematic approach will be indispensable for improving the score in the domain of “Rigor of development”. For the lowest-scoring domain of “Applicability” (46.5%), three of the six guidelines (2018 DELPHI, 2018 IPS, and 2020 EAN) did not address potential resource investment issues in applying recommendations, suggesting that this domain has not been taken seriously by the compilers of current guideline development processes. Obstacles in guideline implementation are the main considerations for clinicians when applying guidelines. To identify the obstacles that may be encountered in the process of guideline application, guideline developers and users can use assessment tools, such as the “Guideline Implementability Appraisal” instrument. In addition, including multidisciplinary experts will facilitate the guideline application process.

The work of Azermai et al. (5) reported results similar to ours. The domains of “Scope and purpose” and “Clarity of presentation” scored best. It is suggested that guideline makers have generally paid attention to the professionalism of the content covered by the guideline and the clarity of the recommendations in the process of guideline formulation. In addition, the “Editorial independence” domain scored lower in the work of Azermai et al., suggesting that this domain has been given attention in guideline development after 2012. Conversely, the “Applicability” domain had the lowest score in both studies. Therefore, the domain of “Applicability” requires greater attention in the development of high-quality guidelines for the management of BPSD in the future.

### Recommendation Extraction From High-Quality CPGs

#### Pharmacological Treatment

##### Psychotic-Like Symptoms Management

Among the four high-quality CPGs, the principles of treatment for psychosis-like symptoms were unanimously recognized. All guidelines suggest that the choice of antipsychotics should be based on the individual risk-benefit ratios of each drug. When the patients with severe agitation and/or aggressiveness that could probably lead to harming themselves or others, the use of atypical antipsychotics is recommended. Furthermore, it is recommended that antipsychotics should be started from a low dose and slowly increased to the effective dose by regularly evaluating individual responses for drugs. After receiving antipsychotic treatment, patients with dementia should be considered about discontinuation of antipsychotics drugs, during experience stable symptoms, or present serious side effects, or their symptoms do not improve.

In terms of drug of choice to treat psychotic-like symptoms in patients with dementia, different from the conclusion of systematic review based on behavioral and psychological symptom management guidelines for dementia reported in 2012, haloperidol (typical antipsychotics) is no longer recommended as a priority drug at the suggestion from two guidelines of 2015 APA and 2020 EAN. In Michel's study, haloperidol was also found to be associated with increased mortality in dementia (16). In recent years, atypical antipsychotics, instead of typical ones, have received increasing attention due to their higher efficacy and fewer adverse events. Jin et al. (17) conducted a Bayesian network meta-analysis and found that atypical antipsychotic quetiapine was as effective as haloperidol, but quetiapine with less adverse events than latter one. Among all the atypical antipsychotic drugs for the treatment of psychotic-like symptoms in dementia, risperidone is recommended as the preferred treatment drug in the 2018 CANCDA and 2020 EAN guidelines. These recommendations are supported by the meta-analysis result, due to comparing the safety of atypical antipsychotics for the management of BPSD, showed that among olanzapine, risperidone, and quetiapine, the incidence of somnolence induced by risperidone was the lowest (18).

It is worth noting that the use of antipsychotics should be with a time limitations. Among the recommended suggestions by the three guidelines of 2015 APA, 2018 CANADA and 2018 NICE, antipsychotic medicine should be gradually reduced or discontinued, when patients with dementia have been treated with antipsychotic drugs up to 3 months, or no significant clinical response founded after 4 weeks of therapy. And during the use of antipsychotic medication or when medication is being tapered, the periodic evaluation for symptoms is necessary which will determine whether patients have receiving an effect therapy or show signs of recurring symptoms. More consistent recommendations from four high-quality CPGs selected in this study, the symptoms of patients with dementia treated with atypical antipsychotics should be evaluated every 4–6 weeks at the beginning of treatment with atypical antipsychotic, every 1–2 weeks at drugs reduction phase, and at least 4 months after drugs withdrawal.

##### Management of Emotional Symptom and Sleep Disorder

Depression is another common clinical symptom in patients with dementia. Among the high-quality guidelines screened in this study, the recommendation in 2018 NICE guideline present against routinely prescribing antidepressants in individuals with mild to moderate dementia. This is an updated and completely contrary to the recommendation of antidepressants in the treatment of depressive symptoms in patients with dementia in the 2012 systematic review (5). The opinions about depression treatment in 2018 NICE guideline were supported by the results from several large randomized, double-blind placebo trials, which antidepressants did not significantly improve depressive symptom, but increase the additional risks in patients with dementia. The cochrane systematic review based on 10 randomized, double-blind placebo trials, showed almost no difference neither in the depression symptom scale nor patients' daily activities and cognitive abilities between the antidepressant group and the placebo group, while more adverse reactions such as dizziness showed in antidepressant treatment than placebo (19). Therefore, antidepressants are not preferential treatment for dementia patients with mild to moderate depression.

Sleep disorders are common clinical problems in dementia, and are associated with significantly increased carer distress and healthcare costs. In term of drug selection for sleep disorders associated with dementia in 2018 NICE guideline, the recommendation against the use of melatonin to manage insomnia in individuals with Alzheimer's disease. McCleery et al. (20) reached similar conclusions by evaluating the efficacy and adverse effects of melatonin, melatonin receptor agonists, trazodone vs. placebo for the treatment of sleep disorders in patients with dementia and showed that melatonin (in doses up to 10 mg) or melatonin receptor agonists were not effective in improving sleep disorders. The 2018 CANADA guideline strongly recommends eliminating antipsychotic use for the treatment of insomnia, such as atypical antipsychotics quetiapine, due to the lack of evidence for the efficacy of antipsychotics in treating insomnia, but rise up the potentially harmful side effects, and high cost of treatment. Therefore, the antipsychotics and melatonin are not recommended attribute to no conclusive evidence for the benefits outweigh the risks.

#### Non-pharmacological Treatment

Non-pharmacological treatments may be suitable for emotional symptoms and sleep disorders. It is considered highly safe and has the advantage of few adverse reactions compared to pharmacological treatment and it includes relaxation, social interaction, sensory therapy (music, aromatherapy), structured activities, sleep hygiene education, psychotherapy and so on. Two guidelines of 2018 NICE and 2018 CANADA unanimously propose that patients with dementia and sleep disorders should receive sleep hygiene education including avoiding caffeine before bed, regular exercise, managing stress, reducing bedroom noise, etc. And the 2018 NICE guideline recommend that psychotherapy should be considered for dementia patients with mild to moderate depression. Thus, the personalized non-pharmaceutical interventions could be adopted as an alternative and effective treatments for depression and sleep disorders in patients with dementia.

### Limitations

The present systematic review had several limitations. First, the AGREE II tool does not provide clear boundaries to distinguish between high-quality and low-quality CPGs. The high-quality criteria defined in this study were referenced from other literature on similar topics. In addition, this study paid more attention to the quality of the guideline formulation process with the AGREE II tool, in term of recommendations put forward in the guideline, and the applicability of the recommendations was not evaluated with the AGREE REX tool, only supplemented by the latest meta-analysis and randomized controlled trial results. Second, although the management of BPSD was in the scope of all guidelines we investigated, the focus of each CPG was different. Specifically, two guidelines (2018 NICE and 2020 EAN) focus more generally on the management of dementia, and there is less focus on the treatment of BPSD. Two other guidelines are mainly concerned with the use and discontinuation of antipsychotics (2015 APA and 2018 CANADA). Therefore, the recommendations for pharmacological and non-pharmacological treatment of BPSD were not exhaustive across the guidelines. In addition, as this study focused on the quality assessment of the guidelines for BPSD and extracted recommendations based on these guidelines, it inevitably ignored some valuable and reference-significant reviews, and thus could not provide comprehensive recommendations for BPSD of different diseases.

### Summary and Outlook

Briefly, strict CPGs can help clinicians provide effective support for the management of BPSD. Based on the AGREE II scores of each field of interest, this review hopes that guideline experts can pay more attention to developing the “Applicability” domain. In addition, as the AGREE II tool is mainly aimed at quality assessment during the guideline development process, to comprehensively screen for high-quality guidelines, the combination of the AGREE II and AGREE REX tools can be considered in the future, where the latter is a supplement to the former and can evaluate the credibility or implementability of guideline recommendations when applied to clinical practice. Future systematic review of CPGs on treatment for BPSD may provide more comprehensive recommendations regarding the treatment, efficacy monitoring, and discontinuation of antipsychotics for BPSD.

## Data Availability Statement

The original contributions presented in the study are included in the article/Supplementary Material, further inquiries can be directed to the corresponding author.

## Author Contributions

HM and XL contributed equally to search data and drafted the manuscript. CW designed the study and revised the manuscription with JL, LJ, and JJ. MZ, QZ, SG, and YC participated in the search for related clinical practice guidelines. AZ, FW, and XZ conducted the literature review.

## Funding

This study was funded by the National Major R&D Projects of China-Scientific Technological Innovation 2030 (No. 2021ZD0201802), National Key R&D Program of China (No. 2017YFC1310103), and Beijing Municipal Administration of Hospitals Clinical Medicine Development of Special Funding Support (No. ZYLX201837).

## Conflict of Interest

The authors declare that the research was conducted in the absence of any commercial or financial relationships that could be construed as a potential conflict of interest.

## Publisher's Note

All claims expressed in this article are solely those of the authors and do not necessarily represent those of their affiliated organizations, or those of the publisher, the editors and the reviewers. Any product that may be evaluated in this article, or claim that may be made by its manufacturer, is not guaranteed or endorsed by the publisher.
