# Supplementary material for: Clinical Practice Guidelines for the Management of Behavioral and Psychological Symptoms of Dementia: A Systematic Review With AGREE II
Source: Front Neurol. 2022 May 25;13:799723. doi: 10.3389/fneur.2022.799723 (PMC9174457; doi:10.3389/fneur.2022.799723)
Supplement: Supplementary file 1 [file Table_1.DOCX]

Supplementary Material

| Contents | | | Included High-quality Clinical Practice Guidelines | | | |
| --- | --- | --- | --- | --- | --- | --- |
|  |  |  | 2015 APA | 2018 CANADA | 2018 NICE | 2020 EAN |
| Pharmacological treatment | Psychotic-like symptoms | Antipsychotic  application | 1. Principles of Application 2. Nonemergency antipsychotic medication should only be used for the treatment of agitation or psychosis in patients with dementia when symptoms are severe, are dangerous, and/or cause significant distress to the patient; (1B) 3. Reviewing the clinical response to nonpharmacological interventions prior to nonemergency use of an antipsychotic medication to treat agitation or psychosis in patients with dementia; (1C) 4. Before nonemergency treatment with an antipsychotic is initiated in patients with dementia, the potential risks and benefits from antipsychotic medication be assessed by the clinician and discussed with the patient (if clinically feasible) as well as with the patient’s surrogate decision maker (if relevant) with input from family or others involved with the patient; (1C) 5. If a risk/benefit assessment favors the use of an antipsychotic for behavioral/psychological symptoms in patients with dementia, treatment should be initiated at a low dose to be titrated up to the minimum effective dose as tolerated; (1B)   (2) Use of Antipsychotics   1. In the absence of delirium, if nonemergency antipsychotic medication treatment is indicated, haloperidol should not be used as a first-line agent; (1B) 2. In patients with dementia with agitation or psychosis, a long-acting injectable antipsychotic medication should not be utilized unless it is otherwise indicated for a co-occurring chronic psychotic disorder; (1B) | 1. Principles of Application 2. Restart antipsychotics at lowest dose possible if resurgence of BPSD with re-trial of deprescribing in 3 months; 3. Review medications that might be worsening symptoms;   (2) Use of Antipsychotics   1. If BPSD relapses and need to restart antipsychotic treatment, consider changing to risperidone, olanzapine, or aripiprazole; | 1. Principles of Application 2. Only offer antipsychotics for people living with dementia who are either: at risk of harming themselves or others or experiencing agitation, hallucinations or delusions that are causing them severe distress; (offer) 3. Before starting antipsychotics, discuss the benefits and harms with the person and their family members or carers (as appropriate); 4. When using antipsychotics: use the lowest effective dose and use them for the shortest possible time and reassess the person at least every 6 weeks, to check whether they still need medication; 5. Use of Antipsychotics 6. Do not offer valproate to manage agitation or aggression in people living with dementia, unless it is indicated for another condition; | 1. Principles of Application 2. Individuals with dementia and agitation and/or aggression should be treated with atypical antipsychotics only after all non-pharmacological measures have been proven to be without benefit or in the case of severe self-harm or harm to others; (weak recommendation) 3. Shared decision-making must be emphasized, and the patient as well as a legal guardian in instances where one has been appointed must be informed about the individual risk–benefit ratio; 4. In instances where treatment with antipsychotics is initiated, starting low with slow up titration to the minimally effective dose or until unacceptable side effects occur; 5. Follow-up for all patients should be planned and a preplanned stop date should be considered since symptoms may remit spontaneously;   (2) Use of Antipsychotics   1. There should be a weak recommendation against treatment of behavioral symptoms in persons with dementia with mild analgesics; (weak recommendation) 2. There should be a weak recommendation for treatment of patients with dementia and agitation/aggressive behavior with modern (atypical) antipsychotics compared to haloperidol when pharmacological treatment of agitation/aggressive behavior is necessary; (weak recommendation) 3. Among modern (atypical) antipsychotics, risperidone may be considered as first-line treatment when pharmacological treatment of agitation/aggressive behavior is necessary; |
|  |  | Antipsychotic Discontinuation | (1) Principles and Measures of Antipsychotic Discontinuation   1. If a patient with dementia experiences a clinically significant side effect of antipsychotic treatment, the potential risks and benefits of antipsychotic medication should be reviewed by the clinician to determine if tapering and discontinuing of the medication is indicated; (1C) 2. In patients with dementia with agitation or psychosis, if there is no clinically significant response after a 4-week trial of an adequate dose of an antipsychotic drug, the medication should be tapered and withdrawn; (1B) 3. In patients with dementia who show adequate response of behavioral psychological symptoms to treatment with an antipsychotic drug, an attempt to taper and withdraw the drug should be made within 4 months of initiation, unless the patient experienced a recurrence of symptoms with prior attempts at tapering of antipsychotic medication; (1C) 4. In patients with dementia whose antipsychotic medication is being tapered, assessment of symptoms should occur at least monthly during the taper and for at least 4 months after medication discontinuation to identify signs of recurrence and trigger a reassessment of the benefits and risks of antipsychotic treatment; (1C) | (1) Principles and Measures of Antipsychotic Discontinuation   1. For adults with BPSD treated for at least 3 months (symptoms stabilized or no response to adequate trial), we recommend the following: taper and stop antipsychotics slowly in collaboration with the patient and caregivers: e.g., 25%-50% dose reduction every 1-2 week; (strong recommendation, moderate-quality evidence) | (1) Principles and Measures of Antipsychotic Discontinuation   1. Stop treatment with antipsychotics: if the person is not getting a clear ongoing benefit from taking them and after discussion with the person taking them and their family members or carers; (as appropriate) | (1) Principles and Measures of Antipsychotic Discontinuation   1. Antipsychotics should be discontinued after cessation of behavioral disturbances and in patients in whom there are side effects;（good practice statement） |
|  | Emotional symptoms | | - | - | 1. Do not routinely offer antidepressants to manage mild to moderate depression in people living with mild to moderate dementia, unless they are indicated for a pre-existing severe mental health problem; | - |
|  | [Sleep](file:///\\Sleep) disorder | | - | 1. Based on the lack of evidence for the efficacy of antipsychotics for treating insomnia, and the potential for harm and high cost, we rated the recommendation to eliminate antipsychotic use for the treatment of insomnia as strong; 2. For adults with primary insomnia treated for any duration or secondary insomnia in which underlying comorbidities are managed, we recommend the following: Stop antipsychotics; tapering is not needed; (good practice recommendation) 3. If the patient has been taking an antipsychotic for a short period of time (e.g., < 6 weeks), stop antipsychotic use immediately. If the patient has been taking the antipsychotic for a longer period of time, consider tapering the dose first before stopping. If there are concerns on the part of either the patient or the prescriber about possible side effects of immediate discontinuation, tapering can also be considered; | 1. Do not offer melatonin to manage insomnia in people living with Alzheimer's disease; | - |
| Non-pharmacological  interventions | Psychotic-like symptoms | | - | 1. Consider interventions such as: relaxation, social contact, sensory (music or aroma-therapy), structured activities and behavioral; 2. Consider environment: e.g., light, noise; 3. If BPSD relapses: Consider non-drug approaches e.g., music therapy, behavioral management strategies); | 1. For people living with dementia who experience agitation or aggression, offer personalized activities to promote engagement, pleasure and interest; (offer) 2. Ensure that people living with dementia can continue to access psychosocial and environmental interventions for distress while they are taking antipsychotics and after they have stopped taking them; | - |
|  | Emotional symptoms | | - |  | 1. For people living with mild to moderate dementia who have mild to moderate depression and/or anxiety, consider psychological treatments; (consider) | - |
|  | [Sleep](file:///\\Sleep) disorder | | - | 1. For those prescribed antipsychotics for the treatment of insomnia, we recommend the following: All patients should be counseled about nonpharmacologic approaches to sleep (so-called sleep hygiene); | 1. For people living with dementia who have sleep problems, consider a personalized multicomponent sleep management approach that includes sleep hygiene education, exposure to daylight, exercise and personalized activities; (consider) | - |

**Supplementary** **Table 1** Recommendations from four included high-quality clinical practice guideline
